# Gonadal response after a single-dose stimulation test with recombinant human chorionic gonadotropin (rhCG) in patients with isolated prepubertal cryptorchidism

**DOI:** 10.1186/s12610-016-0039-2

**Published:** 2016-10-28

**Authors:** Leticia Ribeiro Oliveira, Thais Kataoka Homma, Renata Reis Woloszynek, Vinícius Nahime Brito, Carlos Alberto Longui

**Affiliations:** 1 Pediatric Endocrinology Unit, Pediatrics Department, Irmandade da Santa Casa de Misericórdia de São Paulo, and Santa Casa de São Paulo School of Medical Sciences, Rua Dr. Cesário Mota Jr, 112, Vila Buarque, São Paulo, CEP 01221-020 Brazil; 2 Developmental Endocrinology Unit, University of São Paulo Faculty of Medicine Clinics Hospital - USP, São Paulo, Brazil

**Keywords:** Gonadotrophine chorionique humaine, Cryptorchidie, Testostérone, Hormone antimüllérienne, rhCG recombinante, Human Chorionic Gonadotropin, Cryptorchidism, Testosterone, Anti-Mullerian hormone, Recombinant hCG

## Abstract

**Background:**

The evaluation of prepubertal gonadal Leydig cells secretion requires gonadotropin stimulation. Urinary hCG (human chorionic gonadotropin) is currently unavailable in many countries, however, recombinant hCG (rhCG) can be used. Our aim was to evaluate rhCG-stimulated testicular hormones in a group of patients with cryptorchidism.

**Methods:**

We evaluated 31 prepubertal boys (age range, 0.75–9.0 years) presenting with unilateral (*n* = 24) or bilateral (*n* = 7) cryptorchidism. Patients with other genital abnormalities, previous use of hCG or testosterone or previous surgeries were excluded. Blood samples were obtained at baseline and 7 days after a single subcutaneous dose of rhCG (Ovidrel® 250 mcg) to measure the testosterone, DHT (dihydrotestosterone), AMH (anti-Mullerian hormone), and inhibin B levels.

**Results:**

rhCG stimulation significantly increased testosterone levels from 10 ng/dl to 247.8 ± 135.8 ng/dl, increased DHT levels from 4.6 ± 0.8 to 32.3 ± 18.0 ng/dl, and increased the T/DHT ratio from 2.2 ± 0.4 to 8.0 ± 3.5. There was also a significant increase in inhibin B (from 105.8 ± 65.2 to 132.4 ± 56.1 pg/ml; *p* < 0.05) and AMH levels (from 109.4 ± 52.6 to 152.9 ± 65.2 ng/ml; *p* < 0.01) after the rhCG stimulation.

**Conclusions:**

In this cohort, hormonal responses can be elicited after the rhCG stimulation test, suggesting that rhCG is a promising stimulation test to replace the urinary hCG test during the evaluation of gonadal Leydig cells function. The clinical applicability and adequate performance of rhCG testing must be investigated in future studies.

**Electronic supplementary material:**

The online version of this article (doi:10.1186/s12610-016-0039-2) contains supplementary material, which is available to authorized users.

## Résumé

**Contexte:**

L’évaluation de la sécrétion des cellules gonadiques de Leydig prépubères nécessite une stimulation par les gonadotrophines. La gonadotrophine chorionique humaine (hCG) urinaire est actuellement indisponible dans de nombreux pays; toutefois, l’hCG recombinante (rhCG) peut être utilisée. Notre objectif était d’évaluer les hormones testiculaires sous stimulation par rhCG dans un groupe de patients qui présentaient une cryptorchidie.

**Méthodes:**

Nous avons évalué 31 garçons prépubères (âgés de 0,75 à 9 ans) qui présentaient une cryptorchidie uni (*n*=24) ou bilatérale (*n*=7). Ont été exclus les patients avec d’autres anomalies génitales, ceux qui avaient été traités auparavant par hCG ou testostérone ou par chirurgie. Des échantillons sanguins ont été prélevés au départ et à 7 jours après une unique dose sous cutanée de rhCG (Ovidrel® 250 mcg) pour les mesures des taux de testostérone, dihydrotestostérone (DHT), hormone antimüllérienne (AMH) et inhibine B.

**Résultats:**

La stimulation par rhCG augmente de façon significative les taux de testostérone de 10 ng/dl à 247.8±135.8 ng/dl, de DHT de 4.6±0.8 à 32.3±18.0 ng/dl, et le ratio T/DHT de 2.2±0.4 à 8.0±3.5. Les taux d’inhibine B (de 105.8±65.2 à 132.4±56.1 pg/ml; *p*< 0.05) et d’AMH (de 109.4±52.6 to 152.9±65.2 ng/ml; *p*< 0.01) ont aussi été significativement augmentés après stimulation par rhCG.

**Conclusion:**

Dans cette cohorte, des réponses hormonales ont été obtenues après le test de stimulation par rhCG; ceci suggère que rhCG est un test de stimulation prometteur pour remplacer le test par l’hCG urinaire lors de l’évaluation de la fonction gonadique des cellules de Leydig. L’applicabilité clinique et l’évaluation adéquate de la performance du test par rhCG doivent être étudiées dans de futures études.

## Background

The hypothalamic-pituitary axis is inactivated during childhood, and the testes are functionally quiescent regarding androgen production by Leydig cells. Therefore, during the prepubertal phase, testicular steroidogenesis cannot be evaluated by measuring basal steroid levels; testicular steroidogenesis evaluation can be performed only after recombinant luteinizing hormone (LH) and/or gonadotropin chorionic hormone (hCG) stimulation [1–3].

hCG stimulates the androgen secretion of Leydig cells, allowing the identification of any intra-abdominal testicular tissue in patients with true bilateral cryptorchidism. The hCG test is also used to differentiate between constitutional pubertal delay and hypogonadotropic hypogonadism, as well as to investigate patients with sex differentiation disorders (i.e., by recognising the presence of testicular tissue and enzymatic defects) [1].

Stimulatory tests using chorionic gonadotropin extracted from the urine of pregnant women (uhCG) have long been used to characterise the pattern of gonadal steroid production. At least three test-protocols using intramuscular doses of 100 IU/Kg (maximum of 2000 IU) have been well described: one daily dose for five consecutive days, one dose every 4 days (a total of four injections), and one injection every week for six consecutive weeks [1, 4]; in these protocols a testosterone response higher than 140 ng/dl is considered satisfactory. As in many other countries, uhCG is currently unavailable in Brazil, and it has been replaced by recombinant human chorionic gonadotropin (rhCG) [5–7]. To the best of our knowledge, no previous study of the use of rhCG in children has been published.

In this study, we used rhCG stimulation testing to evaluate gonadal function in a cohort of patients with cryptorchidism.

## Methods

This prospective study included cryptorchid boys during 4 years, evaluating 31 prepubertal boys (mean chronological age, 3.3 years; SD, 2.7 years; range, 0.75–9.0 years) with unilateral (*n* = 24) or bilateral (*n* = 7) cryptorchidism. Patients with any other genital abnormality, previous use of hCG or testosterone or previous surgeries were excluded. After receiving approval from the Institutional Ethics Committee, informed written consent was obtained for each patient from the parents or legal guardians.

Blood samples for the hormonal measurements were obtained at baseline and 7 days after a single subcutaneous dose of rhCG (Ovidrel® 250 mcg).

The following hormonal concentrations were measured at both time points: testosterone (T) (chemiluminescent assay, L2KTW2, Gwynedd, England), dihydrotestosterone (DHT) (radioimmunoassay post-extraction KIPI9900, Louvain La Neuv, Belgium) androstenedione (radioimmunoassay, DSL3800, Prague, Czech Republic), 17-hydroxyprogesterone (17OHP) (radioimmunoassay, KIP1409, Louvain-la-Neuve, Belgium), inhibin B and anti-Mullerian hormone (AMH) (ELISA, Gen II Beckman Coulter Company, TX, USA). Beta-hCG was measured using a post-hCG sample (chemiluminescent assay, Beckman Coulter, Inc. 4300 N. Harbor Blvd., Fullerton, CA, USA). The lower limits of detection for testosterone, DHT, 17OHP, AMH, inhibin B and beta-hCG were 15 ng/dl, 3 ng/dl, 0.11 ng/ml, 4.8 pg/ml, 0.2 ng/ml, and 2.7 mUI/ml, respectively. The intra-assay coefficient of variation (CV) for testosterone, DHT, 17OHP, beta-hCG were 2.0 %, 4.8 %, 5 %, and 1.6 %, respectively, and the limits varied from 2.4 to 2.9 % and 5.2 to 9.0 % for inhibin B and AMH, respectively. The inter-assay CV for DHT was 15 %, and it varied from 4.6 to 7.8 % for AMH.

Statistical analyses were performed using SigmaStat for Windows (version 3.5, SPSS Inc., San Jose, CA, USA). Descriptive results were presented as the means ± standard deviation score (SDS) and percentile. To compare the quantitative variables before and after the rhCG stimulation, a paired *t*-test or Wilcoxon signed-rank test was used according to the distribution patterns. To establish correlations between variables, Pearson’s or Spearman’s coefficient of correlation was calculated. Statistical significance was set at *p* <0.05.

## Results

The 31 prepubertal boys had normal phallus lengths (according to Lee et al. [8]), and no syndromic facies or genital abnormalities. When testosterone levels from bilateral cryptorchid boys (basal testosterone: 10 ± 0.0 ng/dl, rhCG-stimulated testosterone: 253.6 ± 128.0) were compared with testosterone from unilateral cryptorchid boys (basal testosterone: 10 ± 0.0 ng/dl, rhCG-stimulated testosterone: 228.0 ± 169.9), no significant difference was observed (*p* = 0.669). Therefore, both groups are presented as a single group.

Hormonal concentrations were assessed at baseline and 7 days after the rhCG stimulation (Table 1) and (Additional file 1). As expected, we observed normal basal concentrations for all hormone levels in the group of cryptorchidism patients with any other genital abnormality or complications (Table 1).

**Table 1:** Hormonal concentrations at baseline and 7 days after a single subcutaneous dose of human chorionic gonadotropin (rhCG, Ovidrel® 250 mcg)

Hormones	Basal	After rhCG (7 days)
	mean (SD)	mean (SD)
Testosterone (ng/dl)	10.0 (0)	247.8 (135.8)
DHT (ng/dl)	4.6 (0.8)	32.3 (18.0)
T/DHT	2.2 (0.4)	8.0 (3.5)
Inhibin B (pg/ml)	105.8 (65.2)	132.4 (56.1)
AMH (ng/ml)	109.4 (52.6)	152.9 (65.2)
Androstenedione (ng/ml)	0.2 (0.4)	0.3 (0.2)
17OHP (ng/ml)	0.4 (0.3)	0.7 (0.4)
FSH (IU/L)	0.6 (0.4)	0.2 (0.1)
LH (IU/L)	0.1 (0.1)	0.1 (0.2)
Beta hCG (IU/L)	-	15.8 (10.2)

*hCG* human chorionic gonadotropin, *rhCG* recombinant human chorionic gonadotropin, *DHT* dihydrotestosterone hormone, *T/DHT* Testosterone/DHT ratio, *AMH* Anti-Mullerian hormone, *17OHP* 17-hidroxi-progesterone hormone, *FSH* Follicle stimulanting hormone, *LH* Luteinizing hormone, *SD* standard deviation

A significant negative correlation was identified when peak testosterone after rhCG was correlated to the age in which stimulation test was performed (*p* = 0.035; r = 0.38).

Beta hCG levels were detectable during the stimulation test, indicating that the drug was adequately injected. Stimulation with a single subcutaneous dose of rhCG significantly increased testosterone production (paired *t*-test, *p* < 0.001) from 10 ng/dl at baseline to 247.8 ± 135.8 ng/dl post stimulation (Table 1). The testosterone percentile values (ng/dl) were p5(62.9), p10(106.2), p25(131.5), p50(200.0), p75(317.2), p90(436.6) and p95(520.8).

**Fig. 1:**
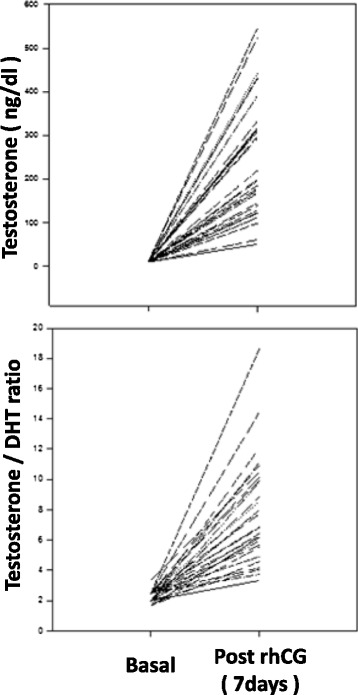
Individual responses of cryptorchid patients (n:31) before and after the stimulatory test with a single subcutaneous dose of recombinant human chorionic gonadotropin (rhCG, Ovidrel® 250 mcg). rhCG: recombinant human chorionic gonadotropin; DHT: dihydrotestosterone hormone

The mean DHT values were 4.6 ± 0.8 ng/dl at baseline and 32.3 ± 18.0 ng/dl after the rhCG stimulation (paired *t*-test, *p* < 0.001) (Table 1). After the hCG stimulation, the T/DHT ratio (Fig. 1) also increased significantly from 2.2 ± 0.4 to 8.0 ± 3.5 (paired *t*-test, *p* < 0.001). Based on the 10^th^ percentile of our results, after 7 days of rhCG stimulation, we defined the following normal inferior response limits for the testosterone levels and T/DHT ratio: 106.2 ng/dl and 4.0, respectively.

There was also a significant increase in inhibin B (from 105.8 ± 65.2 to 132.4 ± 56.1 pg/ml; *p* < 0.05) and AMH levels (from 109.4 ± 52.6 to 152.9 ± 65.2 ng/ml; *p* < 0.01) after the rhCG stimulation. Based on the 10^th^ percentile of our results, we defined the normal lower limits of detection after rhCG stimulation as 63.7 pg/ml and 76 ng/ml for inhibin B and AMH, respectively. There was no significant variation in the steroid precursors, such as the 17OHP and androstenedione levels.

**Fig. 2:**
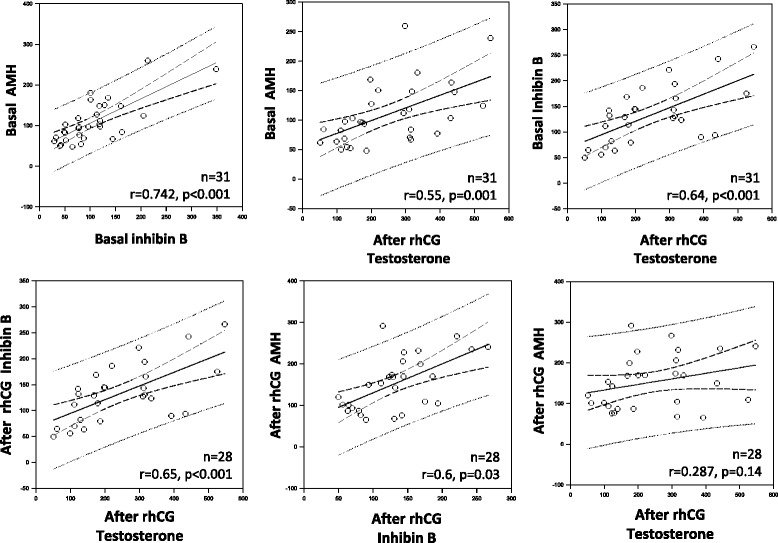
Correlations between the hormonal concentrations in the cryptorchid patients (n:31) at baseline and after the recombinant human chorionic gonadotropin test. rhCG: recombinant human chorionic gonadotropin; AMH: Anti-Mullerian hormone

The correlation between variables revealed positive and significant correlations for the following comparisons: basal AMH and basal inhibin B levels (r = 0.742, *p* < 0.001), basal AMH and 7-day post-rhCG testosterone levels (r = 0.55, *p* = 0.001) basal inhibin B and 7-day post-rhCG testosterone levels (r = 0.64, *p* < 0.001), 7-day post-rhCG inhibin and 7-day post-rhCG testosterone levels (r = 0.65, *p* < 0.001), and 7-day post-rhCG AMH and 7-day post-rhCG inhibin B levels (r = 0.6, *p* = 0.03). No significant correlation was observed when the 7-day post-rhCG AMH level was compared with the 7-day post-rhCG testosterone level (r = 0.287, *p* = 0.14) (Fig. 2).

## Discussion

The hCG stimulation test is useful for several clinical conditions, such as the investigation of a potential androgen insufficiency, detection of testicular tissue undetectable during physical examination, and identification of enzymatic defects of testicular steroidogenesis [9]. In addition, analysing the peak testosterone and DHT secretion levels after hCG, as well its ratio, represent an important tool in the study of patients with abnormal sex differentiation.

The hypothalamic-pituitary-Leydig cells axis is not activated in the prepubertal phase, therefore, the evaluation of Leydig cells function requires pharmacological stimulation with gonadotropins [1, 2]. Until recently, the stimulation test was performed using urinary hCG, but the withdrawal of the medication from the markets in many countries and the availability of the recombinant formulation indicates a need for this new test standardisation.

Isolated cryptorchidism is a condition in which testicular lesion is predominantly observed in tubular Sertoli cells with little or absent involvement of interstitial testosterone-producing Leydig cells. Therefore, at a prepubertal stage, this group of patients would present a potentially normal testosterone response and can be useful as a control group of studies performed in children aiming to investigate abnormalities of sexual differentiation [10–12]. By studying prepubertal cryptorchid patients, we could rule out severe testicular damage at the same time we could recognize potentially adequate values for this specific age, therefore avoiding the ethical issues of performing a testicular stimulation test in normal children.

In this study, by using rhCG (Ovidrel®, 250 mcg, approximately equivalent to 6500 UI), we were able to identify a significant testicular response in prepubertal children after a single subcutaneous dose. Previous standardization protocols employed four to six small hCG doses of 1,000-1,500 UI. This is not possible when using the rhCG as its commercial presentation is a 6500 UI diluted in a reduced volume of 0.5 mL, making impracticable dose fragmentation for this product. The interval of 7 days after rhCG for hormonal assessment was chosen after a pilot study of five individuals in which hormonal data was measured at 3, 5, 7 and 10 days after rhCG; in this subjects testosterone peak response was obtained after 7 days. A previous study in adult healthy men found similar testosterone peak after 250 mcg rhCG and uhCG 5000 UI [6]. In vitro fertilization studies analysing oocyte maturation did not find significant differences in pharmacokinetics between both compounds [5, 7].

Although our results cannot be used as normal reference values, as the rhCG stimulation test was performed in patients with isolated cryptorchidism and with no other associated abnormalities, we suggest the use of our results as an acceptable control response for this test. Our results, which showed similarities in hormonal concentrations between unilateral and bilateral cryptorchidism, align with the results reported by Christiansen et al. [13], who also compared unilateral and bilateral cryptorchid patients with normal controls. An important observation is the negative correlation between age and testosterone during rhCG test. This may indicate a need to adjust the expected testosterone peak response to age.

An increase in inhibin B, which was observed after the hCG stimulation in this study, was also demonstrated in a previous report [13] of cryptorchidism patients treated with intramuscular uhCG in a 3-week stimulation protocol. The authors found a peak inhibin B response of 147 pg/ml, similar to that detected in our patients (129.7 pg/ml). This response can be explained by the fact that in prepubertal testes, Sertoli cells are able to synthesise both inhibin B subunits (alpha and beta B) [13–15]. A different secretion pattern is observed in the late pubertal and adult stages, in which the beta subunit becomes a product of germinal cells, while the alpha subunit is still secreted by Sertoli cells. Therefore, in contrast to observations in children, inhibin B becomes a hormone of combined Sertoli-spermatocyte origin in adults [14, 15]. This finding can explain why the increment of inhibin B is only detected in prepubertal children, such as those included in our study but not in adults [13]. An alternative explanation for the detected inhibin B secretion during rhCG testing is the direct effect of testosterone on prepubertal Sertoli cells. High levels of hCG could bind to FSH receptors and stimulate the hormonal production of Sertoli cells. The possible promiscuous coupling versatility in signalling associated with a single receptor seems to be an intrinsic property of G-protein-coupled receptors [16, 17]. This finding is reinforced by the positive and significant correlation between the testosterone peak and inhibin B peak responses after rhCG stimulation.

Regarding the AMH response, we detected a significant increase in AMH values 7-days after the rhCG application and a positive correlation between the baseline and 7-day AMH levels and post-rhCG testosterone levels. Our results can also be explained in a manner similar to the observations for inhibin B (i.e., by the phase of testicular development in which immature Sertoli cells are stimulated by an acute single dose of rhCG). We may conclude that a parallel increase in inhibin B and AMH suggests that in the prepubertal stage, under acute stimulation, the Sertoli cells retain the capability of proportional secretion of both peptides.

One of the limitations of this study is the measurement of steroid hormones through immunoassays. Recent studies have shown that using mass spectrometry (MS) to measure steroid hormones represents the gold standard when used in the appropriate manner under highly regulated conditions [18], and the immunoassays do not have sufficient sensitivity to detect normal low levels in samples of females and non-stimulated gonads of prepubertal patients [19]. The ability to correlate gonadal function after an acute 7-day stimulation test with gonadal function in adolescents during puberty or in adults (a longitudinal follow-up of these patients is desirable) is not available at this time, and it can represent another limitation of this study.

## Conclusions

Although the protocol and dosage are different for urinary and recombinant hCG we conclude that the 7-day hormonal response after a single subcutaneous dose of rhCG in outpatient clinics is a simple and promising alternative to the urinary hCG test for acute evaluation of prepubertal gonadal secretion. Besides, we have to consider that urinary hCG is currently unavailable in many countries. We show that recombinant hCG triggers a response and provides a proof-of-concept for a future validation of this test. Clinical applicability under distinct conditions must be investigated in further studies.

## Additional file

Additional file 1: Data of hormonal measurements at each time of the assessment. (PDF 235 kb)

## Abbreviations

17OHP: 17-hidroxi-progesterone hormone
AMH: Anti-Mullerian hormone
beta-hCG: β-subunit of hCG
CV: Coefficient of variation
DHT: Dihydrotestosterone hormone
FSH: Follicle stimulanting hormone
hCG: Human chorionic gonadotropin
LH: Luteinizing hormone
P: Percentile
rhCG: Recombinant human chorionic gonadotropin
T: Testosterone
T/DHT: Testosterone/DHT ratio
uhCG: Urinary human chorionic gonadotropin
