# Supplementary material for: Gonadal response after a single-dose stimulation test with recombinant human chorionic gonadotropin (rhCG) in patients with isolated prepubertal cryptorchidism
Source: Basic Clin Androl. 2016 Oct 28;26:13. doi: 10.1186/s12610-016-0039-2 (PMC5084384; doi:10.1186/s12610-016-0039-2)
Supplement: Additional file 1: — Data of hormonal measurements at each time of the assessment. (PDF 235 kb) [file 12610_2016_39_MOESM1_ESM.pdf]

Data of hormonal measurements at each time of the assessment

Case	Type	Age (years)	T (ng/dl) basal	T (ng/dl) peak	DHT (ng/dl) basal	DHT (ng/dl) peak	T/DHT basal	T/DHT peak	Andro (ng/ml) basal	Andro (ng/ml) peak	17 OHP (ng/ml) basal	17 OHP (ng/ml) peak	LH (mU/ml) basal	LH (mU/ml) peak	FSH (mU/ml) basal	FSH (mU/ml) peak	hCG (mU/ml) peak	AMH (ng/ml) basal	AMH (ng/ml peak	Inibina B (pg/ml) basal	Inibina B (pg/ml) peak
1	U	0,83	10	122,0	5,0	30,0	2,0	4,1	0,05	0,50	0,3	0,8	0,1	0,2	1,2	0,3	10,2	68,5	75,8	87,6	141,6
2	U	0,91	10	186,0	5,0	21,0	2,0	8,9	0,20	0,10	0,3	0,3	0,1	0,1	0,3	0,1	14,1	47,9	86,9	65,0	79,3
3	U	1,00	10	431,0	5,0	na	2,0	na	0,05	0,20	0,1	na	0,1	0,1	0,2	0,1	32,2	103,3	na	118,4	na
4	U	1,00	10	220,0	4,0	32,0	2,5	6,9	0,80	0,50	0,3	0,3	0,1	0,1	0,1	0,1	5,7	150,5	169,7	128,7	186,1
5	U	1,00	10	433,0	6,0	44,0	1,6	9,8	0,05	0,05	0,2	0,3	0,1	0,9	0,8	0,1	48,3	163,7	149,6	101,0	93,6
6	U	1,08	10	442,0	4,0	90,0	2,5	4,9	0,30	0,10	0,2	1,0	0,1	0,1	0,4	0,1	20,8	147,7	235,0	160,7	242,6
7	U	1,08	10	334,0	4,0	49,0	2,5	6,8	0,05	0,05	0,4	0,6	0,1	0,2	0,4	0,1	10,4	180,4	168,9	100,8	123,2
8	U	1,08	10	298,0	6,0	65,0	1,6	4,6	0,15	0,15	0,2	0,3	0,1	0,1	0,7	0,1	6,2	259,8	266,6	213,7	221,2
9	U	1,08	10	547,0	4,0	55,0	2,5	9,9	0,10	0,80	0,2	1,2	0,3	0,1	0,1	0,1	21,2	238,9	240,8	348,4	266,3
10	U	1,66	10	294,0	5,0	na	2,0	na	0,05	0,60	0,1	2,0	0,2	0,1	0,3	0,1	18,2	111,7	na	119,5	na
11	U	2,33	10	145,0	4,0	na	2,5	na	0,50	0,10	0,8	0,5	0,1	0,2	0,3	0,4	29,0	102,9	na	51,0	na
12	U	2,50	10	168,0	4,0	45,0	2,5	3,7	0,05	0,10	0,2	0,6	0,2	0,1	1,0	0,2	22,4	97,1	168,2	96,3	128,8
13	U	2,75	10	123,0	5,0	37,0	2,0	3,3	0,15	0,60	0,5	0,7	0,2	0,2	0,9	0,2	19,2	97,5	142,5	119,7	131,8
14	U	2,83	10	112,0	5,0	14,0	2,0	8,0	0,05	0,15	1,0	1,0	0,1	0,1	0,5	0,4	10,1	49,8	93,3	40,6	69,7
15	U	3,00	10	318,0	4,0	22,0	2,5	14,5	0,15	0,30	0,2	0,6	0,1	0,1	1,2	0,2	17,4	148,4	231,8	118,3	165,4
16	U	3,08	10	179,0	5,0	30,0	2,0	6,0	0,10	0,30	1,5	0,4	0,1	0,1	0,3	0,2	10,7	93,4	291,4	77,4	114,0
17	U	4,00	10	310,0	5,0	39,0	2,0	7,9	0,15	0,20	0,5	0,9	0,1	0,2	0,6	0,1	21,6	118,2	173,4	76,6	127,1
18	U	4,83	10	197,0	4,0	18,0	2,5	10,9	0,15	0,40	0,3	0,4	0,2	0,1	0,5	0,1	13,3	168,6	227,6	135,4	144,5
19	U	5,00	10	111,0	4,0	13,0	2,5	8,5	0,05	0,05	0,2	0,5	0,2	0,2	0,8	na	6,5	82,4	153,1	50,5	111,4
20	U	5,00	10	139,0	4,0	18,0	2,5	7,7	0,30	0,40	0,3	0,6	0,1	0,1	0,3	0,1	18,4	52,3	86,4	42,4	63,3
21	U	6,41	10	392,0	4,0	21,0	2,5	18,7	0,05	0,40	1,1	1,1	0,2	0,1	1,4	0,4	6,6	77,2	65,2	78,7	89,8
22	U	7,00	10	99,0	6,0	16,0	1,6	6,2	0,05	0,20	0,8	0,9	0,1	0,1	0,2	0,2	9,2	63,6	101,5	52,3	55,8
23	U	9,00	10	312,0	na	28,0	na	11,1	0,05	0,70	0,6	1,0	0,1	0,1	0,6	0,1	11,6	70,5	206,0	32,7	143,1
24	U	9,00	10	174,0	5,0	27,0	2,0	6,4	0,10	0,30	0,9	2,1	0,2	0,2	0,8	0,2	13,4	96,0	199,3	78,2	168,5
25	B	0,75	10	525,0	3,0	50,0	3,3	10,5	0,10	0,15	0,1	0,6	0,4	0,1	1,3	0,1	7,3	124,1	109,0	205,1	175,0
26	B	0,75	10	315,0	4,0	31,0	2,5	10,2	0,30	0,10	0,5	0,5	0,1	0,1	0,2	0,1	9,6	66,8	104,4	144,6	193,8
27	B	0,83	10	315,0	5,0	26,0	2,0	12,1	0,05	0,50	0,2	0,9	0,3	0,1	1,4	0,1	12,7	83,9	67,5	162,7	130,6
28	B	1,00	10	129,0	4,0	31,0	2,5	4,2	0,10	0,60	0,2	0,4	0,1	0,1	1,0	0,5	12,0	54,3	76,3	82,1	82,0
29	B	5,00	10	51,0	4,0	9,0	2,5	5,7	0,05	0,15	0,4	0,5	0,1	0,1	0,5	0,3	4,5	61,6	119,8	29,2	49,6
30	B	7,25	10	61,0	6,0	11,0	1,6	5,5	0,40	0,40	0,5	0,3	0,1	0,1	0,9	0,2	6,4	84,2	100,8	48,9	64,4
31	B	9,00	10	200,0	4,0	32,0	2,5	6,3	2,10	0,50	0,3	0,9	0,2	0,1	0,9	0,1	39,1	127,4	169,4	113,1	144,0

hCG: human chorionic gonadotropin; basal: basal levels; peak: peak levels obtainned 7 days after a subcutaneous single dose of recombinant hCG (rhCG, Ovidrel® 250 mcg); U: unilateral; B: bilateral; na: not applicable; T: testosterone; DHT: dehydrotestosterone; Andro: androstenedione; 17 OHP: 17-hydroxiprogesterone; LH: luteinizing hormone; FSH: follicle stimulating hormone.
